# Draft Genome Sequence of an Active Heterotrophic Nitrifier-Denitrifier, *Cupriavidus pauculus* UM1

**DOI:** 10.1128/genomeA.00028-18

**Published:** 2018-02-08

**Authors:** Catherine Putonti, Nathaniel Polley, Domenic Castignetti

**Affiliations:** a Department of Biology, Loyola University Chicago, Chicago, Illinois, USA; b Bioinformatics Program, Loyola University Chicago, Chicago, Illinois, USA; c Department of Computer Science, Loyola University Chicago, Chicago, Illinois, USA

## Abstract

Here, we present the draft genome sequence of *Cupriavidus pauculus* UM1, a metal-resistant heterotrophic nitrifier-denitrifier capable of synthesizing nitrite from pyruvic oxime. The size of the genome is 7,402,815 bp with a GC content of 64.8%. This draft assembly consists of 38 scaffolds.

## GENOME ANNOUNCEMENT

The genus *Cupriavidus* is composed of Gram-negative peritrichously flagellated rods with oxidative metabolisms. Members may be chemoheterotrophic or chemolithotrophic, and resistance among them to various metals is widespread. Species are found in soil and as human clinical specimens (1); the species *C. pauculus* has been metabolically characterized (2).

*C. pauculus* UM1, isolated from an agricultural soil sample in Hadley, MA, USA, is a phenol-degrading (3) copper- and nickel-resistant heterotrophic nitrifier (3) and denitrifier (4), capable of oxidizing both the carbon and nitrogen of pyruvic oxime (3). The enzyme responsible for pyruvic oxime oxygenation, pyruvic oxime dioxygenase (POD), has been cloned, characterized, and noted as a class II adolase (5). Recent work (D. Castignetti, unpublished data) has indicated that the POD of *C. pauculus* UM1 has significant nucleotide (75%) and amino acid homologies (85%) to the POD of the *Alcaligenes faecalis* isolate of Tsujino et al. (5).

*C. pauculus* UM1 genomic DNA was isolated as described previously (3) after being grown in nutrient-enriched tryptic soy broth medium using the Wizard Genomic DNA purification kit (Promega, Madison, WI, USA) per the manufacturer’s directions. Genomic DNA concentration was determined using the Qubit fluorimeter. Library preparation for Illumina sequencing was performed at the Loyola University Chicago Genomics Facility (Maywood, IL, USA) using a Nextera XT DNA library preparation kit. The library was sequenced on the MiSeq sequencer (Illumina) using the MiSeq version 2 reagent kit (500 cycles). The run produced 1,266,809 paired-end reads in total. Genomic DNA was also sequenced using the PacBio RS II platform. PacBio library preparation and sequencing were performed at the Yale Center for Genomic Analysis (New Haven, CT, USA). Library preparation was conducted by selecting fragments 3 to 20 kb in size. The run produced 31,552 reads ranging in size from 35 to 44,849 bp.

Illumina reads were trimmed using the tool sickle (https://github.com/najoshi/sickle). PacBio reads were processed using the Hierarchical Genome Assembly Process (HGAP) (6). Trimmed Illumina reads and polished PacBio assemblies were then assembled together using SPAdes version 3.11.1 (7), which produced 142 contigs. Coverage was evaluated using BBMap (http://sourceforge.net/projects/bbmap/); contigs with a coverage of less than one were removed from further consideration. A final set of 38 scaffolds was identified, varying in size from 2,493 bp to 1.05 Mb, with an *N*_50_ of 320,744 bp. Integrating the Illumina and PacBio reads together greatly increased the *N*_50_ score (e.g., for the PacBio assembly, *N*_50_ = 42,691 bp). Sequence coverage was greater than 30× for each sequencing method employed. The genome size was 7,402,815 bp with a GC content of 64.77%. Annotations were produced by the NCBI Prokaryotic Genome Annotation Pipeline (8). Six rRNAs, 59 tRNAs, and 6,473 protein-coding sequences were detected.

### Accession number(s).

The draft whole-genome project for *C. pauculus* UM1 has been deposited at DDBJ/EMBL/GenBank under the accession number PJRP00000000. Raw sequence reads are deposited at DDBJ/EMBL/GenBank under the accession numbers SRR6382437 and SRR6382438.
